# Clinicopathologic characteristics and prognostic analysis of monoclonal gammopathy of renal significance (MGRS) in patients with IgM monoclonal gammopathy: a case series

**DOI:** 10.1038/s41598-022-21152-0

**Published:** 2022-10-10

**Authors:** Jing Liu, Dandan Liang, Shaoshan Liang, Feng Xu, Xianghua Huang, Song Jiang, Jinhua Hou

**Affiliations:** National Clinical Research Center of Kidney Diseases, Jinling Hospital, Jinling Clinical Medical College of Nanjing Medical University, 305 East Zhongshan Road, Nanjing, China

**Keywords:** Nephrology, Kidney diseases

## Abstract

Monoclonal gammopathy has emerged as an important cause of renal injury. Since the clinicopathologic features related to monotypic monoclonal gammopathy of renal significance with IgM monoclonal gammopathy (IgM-MGRS) are poorly described and it is uncertain if intervention improves renal survival and mortality, we report a series of such patients, characterizing their clinicopathologic spectrum and outcomes. We retrospectively analyzed 38 patients referred to one medical center between 2009 and 2019 with detectable serum monoclonal IgM by immunofixation, performance of a bone marrow biopsy and kidney biopsy-proven MGRS. Of the 38 patients identified, about half patients were amyloidosis, followed by cryoglobulinemic glomerulonephritis. Patients were divided into two groups on the basis of their kidney pathology: amyloid and non-amyloid. Patients with non-amyloidosis were more likely to have renal dysfunction, hematuria, anemia and hypocomplementemia and κ light chain was predominant in this sub-group. Amyloid patients were more often treated with chemotherapy than the non-amyloid patients (*P* = 0.002). There were no significant differences between amyloid and non-amyloid patients in mortality (48% vs 29%,* P* = 0.467) and incidence of ESRD (19% vs 59%,* P* = 0.103). The incidence of ESRD was lower in patients treated with chemotherapy and/or ASCT, compared to those without chemotherapy (25% vs 57%, *P* = 0.049), and it was also lower in the hematologic responders than non-responders (10% vs 40%, *P* = 0.047). Our study confirmed a diverse variety of clinicopathological features and outcomes in patients with IgM-MGRS. Chemotherapy and/or ASCT and deep hematologic responses might improve renal prognosis.

## Introduction

Monoclonal gammopathy of renal significance (MGRS) was first introduced in 2012 to acknowledge B cell or plasma cell proliferative disorders that produce monoclonal immunoglobulin or of incomplete immunoglobulin causing renal injury in the absence of hematologic malignancy by the International Kidney and Monoclonal Gammopathy Research Group (IKMG)^1^. Upon progression, the hematologic status of most individuals with IgG, IgA, or free light chain (FLC) MGRS progress into multiple myeloma (MM) or systemic light chain amyloidosis, while most individuals with IgM MGRS tend to develop Waldenström macroglobulinemia (WM) or other lymphoproliferative disorders. Furthermore, it is recommended that IgG, IgA, or FLC associated MGRS should be treated with as per the treatment algorithm for MM, and IgM associated MGRS should follow the treatment algorithm for WM^2,3^. Therefore, MGRS can be separated into two distinct groups, non-IgM MGRS, including IgG, IgA, and kappa or lambda FLC MGRS, and IgM MGRS. The main heavy chain type of M protein in MGRS patients with detectable M protein is usually IgG or IgA. In about 7% of patients, MGRS is associated with an underlying IgM described in small series by our groups^4^. Given its rarity, IgM MGRS remains poorly studied. To contribute to the knowledge base, we reported a retrospective IgM MGRS case series study from our single institute and analyzed the clinicopathologic characteristics of IgM MGRS and its prognosis.

## Results

### Demographics, clinical and renal characteristics at biopsy

We identified 38 MGRS patients, with detectable serum monoclonal IgM by immunofixation who had both a bone marrow and a kidney biopsy. Of these patients, 55% (21 patients) were amyloidosis, including 2 patients accompanied with diabetic nephropathy. Seventeen patients (45%) were non-amyloidosis, including 12 patients (32%) with cryoglobulinemic glomerulonephritis (GN), 3 patients (8%) with proliferative GN with monoclonal IgM deposits, 1 patient (3%) with light-chain deposition disease (LCDD), and 1 patient (3%) with C3 GN. We divided the patients into two groups: amyloid and non-amyloid (Table 1). Renal Histopathological characteristics was shown in Supplementary Table S1.

**Table 1:** Demographics and characteristics at kidney biopsy of patients with MGRS with IgM Monoclonal Gammopathy.

Characteristics	Total	Amyloid	Non-amyloid	*P*-value
N	38	21	17	
Age (IQR), years	62 (52–68)	63 (58–69)	61 (50–66)	0.352
Sex, men/women	26/12	14/7	12/5	0.796
Hypertension, n (%)	18 (47)	3 (14)	15 (88)	< 0.001
Hematuria, n (%)	19 (50)	5 (24)	14 (82)	0.001
Serum creatinine (IQR), mg/dl	1.31 (0.79–2.04)	0.89 (0.78–1.31)	2.00 (1.51–2.87)	< 0.001
eGFR (IQR), ml/min per 1.73 m^2^	58.4 (32.4–83.9)	81.9 (58.4–93.5)	35.3 (19.4–48.5)	0.001
Kidney impairment, n (%)	19 (50)	5 (24)	14 (82)	0.001
Edema, n (%)	32 (84)	16 (76)	16 (94)	0.197
Proteinuria (IQR), g/d	4.21 (2.05–7.24)	4.21 (2.62–6.08)	4.24 (1.14–8.78)	0.774
Serum albumin (IQR), g/l	29.7 (25.4–35.5)	28.9 (23.0–34.0)	30.6 (27.8–39.6)	0.179
Nephrotic syndrome, n (%)	16 (42)	10 (48)	6 (35)	0.521
Hemoglobin (IQR), g/l	113 (100–125)	121 (109–133)	102 (77–112)	0.001
Anemia, n (%)	18 (47)	5 (24)	13 (77)	0.003
Light-chain type, n (%)				0.025
κ	21 (55)	8 (38)	13 (76)	
λ	17 (45)	13 (62)	4 (24)	
Abnormal serum κ/λ FLC ratio, n (%)	18 (47)	10 (48)	8 (47)	1
Low serum C3, n (%)	15 (39)	1 (6)	14 (82)	< 0.001
Low serum C4, n (%)	7 (18)	0	7 (41)	0.007
Serum IgM (IQR), g/l	5.98 (2.87–10.88)	8.22 (3.97–15.10)	3.24 (2.74–6.96)	0.045
Renal disease duration before renal biopsy (IQR), months	5.2 (2.9–14.0)	4.6 (2.1–7.5)	9.6 (3.3–22.5)	0.064
Timing of kidney biopsy, n (%)				0.055
Kidney biopsy before bone marrow biopsy	8 (21)	7 (33.3)	1 (5.9)	
Kidney biopsy within 1 week of bone marrow biopsy	20 (53)	11 (52.4)	9 (52.9)	
Kidney biopsy after bone marrow biopsy	10 (26)	3 (14.3)	7 (41.2)	

There was a total of 26 (68%) men and 12 (32%) women. The median age of the patients at kidney biopsy was 62 years old (IQR 52–68). There was no significant difference in the distribution of gender and the age at diagnosis between the amyloid and non-amyloid groups. The median time from disease onset to renal biopsy was 5.2 months (IQR 2.9–14.0). Renal disease duration before renal biopsy seemed longer in the non-amyloid group; however, there was no significant difference between the two groups (*P* = 0.064).

The median time between kidney and bone marrow biopsy was 0 day (IQR − 5 to 8). Bone marrow biopsy was performed > 1 week before kidney biopsy in 26%, whereas 21% had their kidney biopsy first. Both biopsies were performed within a week of each other in 53%. Six (16%) patients had their kidney biopsy > 1 month before the bone marrow biopsy, and 2 (5%) patients had their bone marrow biopsy > 1 month before the kidney biopsy. In the amyloid group, 33% had their kidney biopsy first and 14% had their bone marrow biopsy first, while 6% had their kidney biopsy first and 41% had their bone marrow biopsy first in the non-amyloid group, which was not significant (*P* = 0.055).

The median proteinuria was 4.21 g/d (IQR 2.05–7.24), and mean serum albumin was 30.04 ± 7.27 g/L. Sixteen (42%) patients had nephrotic syndrome. There was no significant difference in the percentage of nephrotic syndrome between the two groups. The median serum creatinine was 1.31 mg/dl (IQR 0.79–2.04) with a mean eGFR of 58.90 ± 31.08 ml/min/1.73 m^2^. Kidney impairment was present in 53% at the time of kidney biopsy. Hypertension was found in 47% of patients. Nineteen patients (50%) had microscopic hematuria. Compared with non-amyloid patients, amyloid patients had a lower frequency of hypertension and hematuria, and better baseline renal function (higher eGFR) and lower frequency of renal insufficiency. One patient with proliferative GN with monoclonal IgM deposits was on dialysis at the time of renal biopsy, and continue dialysis after treatment.

λ light chain was more common in patients with amyloid-related glomerulopathy (62%), but κ was more common in patients with nonamyloid-related glomerulopathy (76%; *P* = 0.025). The median κ–λ ratio was 0.88 (IQR 0.35–4.33). Abnormal serum free light chain ratio was noted in 18 (47%) patients. Serum IgM level at kidney biopsy was 5.98 g/l (IQR 2.87–10.88). Compared with non-amyloid patients, serum IgM levels at kidney biopsy were higher in amyloid group (*P* = 0.045).

The median hemoglobin at kidney biopsy was 11.3 g/dl (IQR 10.0–12.5), with 18 (47%) patients with anemia. Eight patients (21%) had low serum C3 level and normal serum C4 level including 1 amyloidosis patient, 3 cryoglobulinemic GN patients, 2 proliferative GN with monoclonal IgM deposits patients, 1 LCDD patient, and 1 C3 GN patient. Seven patients (18%) had low serum C3 level and low serum C4 level including 5 cryoglobulinemic GN patients and 2 proliferative GN with monoclonal IgM deposits patients. No patient had low serum C4 level and normal serum C3 level. Non-amyloid patients had a higher frequency of anemia (*P* = 0.003), and a higher frequency of low serum C3 (*P* < 0.001) and C4 (*P* = 0.007), than those with amyloidosis.

In the 21 amyloid patients, thirteen (62%) patients had cardiac involvement, and three (14%) patients had liver involvement. Eighteen (47%) patients had Hypertension, whereas one patient had decreased blood pressure. In the 8 patients with cryoglobulinemic GN, one had nerve involvement, and two patients with type 2 cryoglobulinemia were positive for hepatitis B serology. Six (16%) patients had diabetes, including three patients with amyloid, two patients with cryoglobulinemic GN, and one patient with proliferative GN with monoclonal IgM deposits.

### Treatment

Sixty-three percent were treated with chemotherapy. Bortezomib-based chemotherapy was available for 11 (29%) patients, including nine patients with amyloidosis, one patient with cryoglobulinemic GN and one patient with proliferative GN with monoclonal IgM deposits. Cyclophosphamide-based chemotherapy was used in five (13%) patients, including three patients with amyloidosis, and two patients with cryoglobulinemic GN. Six (16%) patients received thalidomide, including five patients with amyloidosis, and one patient with proliferative GN with monoclonal IgM deposits. One patient with amyloidosis was treated with rituximab alone, and one patient with cryoglobulinemic GN was treated with rituximab and chemotherapy. Seven patients (18%) were treated with corticosteroids with or without immunosuppressive agents, including one patient with amyloidosis, 5 patients with cryoglobulinemic GN, and one patient with C3 GN. Plasmapheresis was used primarily in 2 patients with cryoglobulinemic GN. Seven patients (18%) only received supportive treatment, including two patients with amyloidosis, three patients with cryoglobulinemic GN, one patients with proliferative GN with monoclonal IgM deposits, and one patient with LCDD. Since MGRS was first described in 2012, we analyzed the percentage of treated MGRS patients and showed no difference before and after January 1st 2013 (4/5, 80% vs 26/32, 81%, *P* = 1). The five patients treated before January 1st 2013 included three patients with amyloidosis and two patients with cryoglobulinemic GN. Autologous stem cell transplant (ASCT) as second-line treatment was used in four patients, all with amyloid-related glomerulopathy.

Although analysis is limited by small numbers of patients with no chemotherapy (n = 14), including 7 patients receiving corticosteroids with or without immunosuppressive agents and 7 patients receiving supportive treatment, this sub-group was older (median age of 64 years) compared to the total cohort (median age of 62 years). Kidney impairment was found in 71% of patients. Patients with no chemotherapy had lower eGFR and compared with those with chemotherapy and/or ASCT (*P* = 0.058). In this sub-group, patients had higher incidence of hypertension (71% vs 33%, *P* = 0.042) and higher incidence of anemia (79% vs 29%, *P* = 0.006), compared with patients treated with chemotherapy and/or ASCT. However, cardiac involvement was lower (14% vs 50%, *P* = 0.039). Supplementary Table S2 contains detailed baseline characteristics of patients with no chemotherapy.

Amyloid patients were more often treated with chemotherapy than the non-amyloid patients (*P* = 0.002) (Table 2). About 86% amyloid patients received chemotherapy, and bortezomib-based chemotherapy was most common therapy in amyloid group, followed by thalidomide-based chemotherapy. Only 6 (35%) non-amyloid patients were treated with chemotherapy.

**Table 2:** Treatment and outcomes of patients with MGRS with IgM Monoclonal Gammopathy.

	Total	Amyloid	Non-amyloid	*P*-value
N	38	21	17	
**Treatment, n (%)**				0.025
Supportive	7 (18)	2 (10)	5 (29)	
Corticosteroids with or without immunosuppressant	7 (18)	1 (5)	6 (35)	
Bortezomib-based chemotherapy	11 (29)	9 (43)	2 (12)	
Rituximab-based chemotherapy	2 (5)	1 (5)	1 (6)	
Thalidomide-based chemotherapy	6 (16)	5 (24)	1 (6)	
Cyclophosphamide-based chemotherapy	5 (13)	3 (14)	2 (12)	
Chemotherapy*, n (%)	24 (63)	18 (86)	6 (35)	0.002
**ASCT as second-line treatment, n (%)**	4^$^	4	0	
**Plasmapheresis, n (%)**	2^#^	0	2	
**Hematologic response, n (%)**				
Complete response	5 (13)	4 (19)	1 (6)	
Very good partial response	5 (13)	5 (24)	0	
Partial response	3 (8)	3 (14)	0	
Stable disease	2 (5)	1 (5)	1 (6)	
Progression	5 (13)	3 (14)	2 (12)	
Not accessible	18 (47)	5 (24)	13 (76)	
**Kidney outcomes, n (%)**				
Stable/improved	18 (47)	11 (52)	7 (41)	
Worsened	6 (16)	6 (29)	0	
ESRD	14 (37)	4 (19)	10 (59)	0.103^&^
**Death, n (%)**	15 (39)	10 (48)	5 (29)	0.467^&^

*Chemotherapy included bortezomib-based chemotherapy, rituximab-based chemotherapy, thalidomide-based chemotherapy, and cyclophosphamide-based chemotherapy.

^$^ASCT were used in four patients, including two patients followed cyclophosphamide-based chemotherapy, and two patients followed bortezomib-based chemotherapy.

^#^These two patients were also treated with corticosteroids.

^&^Log-rank test was used to compare survival between amyloid and non-amyloid patients.

### Outcomes

Hematologic response was assessable in 53% of patients. Complete response (CR) was noted in 13% of patients and very good partial response (VGPR) was noted in 13% of patients. CR was achieved in four patients with amyloidosis and one patient with cryoglobulinemic GN, and VGPR was achieved in five patients with amyloidosis. Three patients with amyloidosis achieved partial response, which accounted for 8%. Three of the five patients with CR received rituximab-based chemotherapy, one received rituximab-based chemotherapy followed by ASCT and one received thalidomide-based chemotherapy. Two of the five patients with VGPR received cyclophosphamide-based chemotherapy followed by ASCT, one received rituximab-based chemotherapy followed by ASCT, one received rituximab-based chemotherapy, and one received thalidomide-based chemotherapy. Responses by kidney lesion groups are listed in Table 2.

At the time of censor, response to chemotherapy and/or ASCT was not yet evaluable in four patients. Of the 14 treated with no chemotherapy and/or ASCT, one had a CR response, eight had a < VGPR response, and five was not yet evaluable. Of the 20 response evaluable lines of chemotherapy and/or ASCT, there were 10 ≥ VGPR vs. 10 < VGPR achieved (Table 3). At the time of diagnosis, the median age of patients who achieved ≥ VGPR was 59 (IQR 50–68) compared to 62 (IQR 57–69) in patients with < VGPR. The median eGFR of ≥ VGPR patients at diagnosis was 81.2 ml/min per 1.73 m^2^ (IQR 61.8–98.5) versus 77.3 ml/min per 1.73 m^2^ (IQR 26.6–86.0) in patients with < VGPR. The patients with response had shorter renal disease duration before renal biopsy, compared to non-responders (*P* = 0.035). There was no significant difference in the mortality between the responders and non-responders (30% vs 40%, *P* = 0.123, Supplementary Fig. S1A). ESRD was lower in the responders than non-responders (10% vs 40%, *P* = 0.047, Supplementary Fig. S1B).

**Table 3:** Compared baseline characteristics and treatment of responders (≥ VGPR) and non-responders (< VGPR).

Characteristics	Responders (≥ VGPR)	Non-responders (< VGPR)	*P*-value
N	10	10	
Renal pathological features			
Amyloid/Non-amyloid	9/1	7/3	0.582
Age (IQR), years	59 (50–68)	62 (57–69)	0.315
Sex, men/women	7/3	5/5	0.650
Hypertension, n (%)	3 (30)	3 (30)	1
Cardiac involvement, n (%)	7 (70)	3 (30)	0.179
Liver involvement, n (%)	1 (10)	1 (10)	1
Hematuria, n (%)	4 (40)	4 (40)	1
Serum creatinine (IQR), mg/dl	0.79 (0.73–1.26)	0.93 (0.79–2.08)	0.218
eGFR (IQR), ml/min per 1.73 m^2^	81.2 (61.8–98.5)	77.3 (26.6–86.0)	0.353
Kidney impairment, n (%)	2 (20)	4 (40)	0.628
Edema, n (%)	7 (70)	8 (80)	1
Proteinuria (IQR), g/d	5.70 (2.42–8.46)	2.75 (1.03–3.96)	0.079
Serum albumin (IQR), g/l	28.6 (25.2–37.0)	32.3 (28.1–39.3)	0.280
Nephrotic syndrome, n (%)	6 (60)	1 (10)	0.057
Hemoglobin (IQR), g/l	132 (114–143)	114 (106–122)	0.075
Anemia, n (%)	2 (20)	3 (30)	1
Light-chain type, n (%)			1
κ	4 (40)	5 (50)	
λ	6 (60)	5 (50)	
Abnormal serum κ/λ FLC ratio, n (%)	6 (60)	4 (40)	0.656
Low serum complement, n (%)	2 (20)	3 (30)	1
Serum IgM (IQR), g/l	5.96 (1.52–11.70)	7.43 (3.80–11.90)	0.579
Renal disease duration before renal biopsy (IQR), months	2.9 (1.3–6.6)	6.9 (3.4–17.4)	0.035
Treatment, n (%)			0.836
Bortezomib-based chemotherapy	6 (60)	4 (40)	
Rituximab-based chemotherapy	0	1 (10)	
Thalidomide-based chemotherapy	2 (20)	3 (30)	
Cyclophosphamide-based chemotherapy	2 (20)	2 (20)	

**Figure 1:**
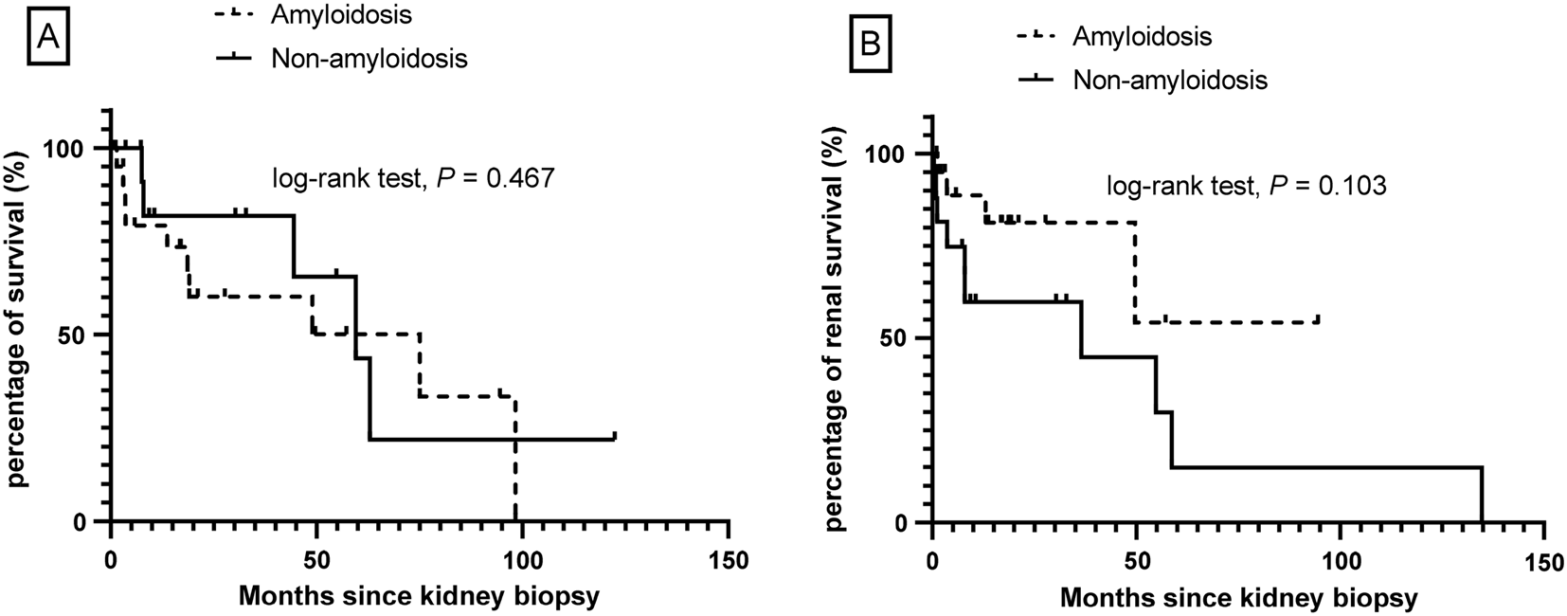
(**A**) Overall survival of IgM-MGRS in patients with IgM Monoclonal Gammopathy from time of kidney biopsy. (**B**) Kidney survival of IgM-MGRS in Patients with IgM Monoclonal Gammopathy from the time of kidney biopsy.

**Figure 2:**
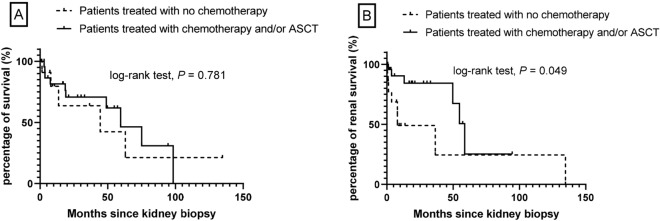
(**A**) Comparing survival of IgM-MGRS patients treated with or without chemotherapy and/or ASCT. (**B**) Comparing renal survival of IgM-MGRS patients treated with or without chemotherapy and/or ASCT.

The mean follow-up time after renal biopsy was 29.0 ± 32.0 months (median: 17.8; IQR 3.6–49.1). The median follow-up time after renal biopsy was 18.6 months (IQR 3.6–49.3) in amyloid group and 10.6 months (IQR 3.6–49.3) in non-amyloid group, but without significant difference between the two groups (*P* = 0.908). At the end of follow-up, 15 patients (39%) died, and median overall survival from the time of kidney biopsy was 59.6 months (95% CI 41.5, 77.6). Amyloid patients seemed have higher mortality (48% vs 29%,* P* = 0.467), compared with non-amyloid patients, although there was no statistical significance between the two groups (Fig. 1A). Cause of death for most patients with amyloidosis was unknown. For five of patients in non-amyloid group, infection (n = 2) and unknown (n = 3) were among the causes of death. Ten (42%) patients treated with chemotherapy and/or ASCT died and five (36%) patients with no chemotherapy died (*P* = 0.781, Fig. 2A).

Kidney parameters improve or stabilized in 47% of patients and worsened in 16% of patients. Fourteen patients (37%) went on to end stage renal disease (ESRD), and median kidney survival from the time of kidney biopsy was 54.8 months (95% CI 29.9, 79.6). Amyloid patients seemed have lower incidence of ESRD (19% vs 59%,* P* = 0.103), compared with non-amyloid patients; however, there was no statistical significance between the two groups (Fig. 1B). In the non-amyloid group, six patients with cryoglobulinemic GN, three patients with proliferative GN with monoclonal IgM deposits and one patient with C3 GN reached ESRD. ESRD was higher in patients with no chemotherapy, compared to those treated with chemotherapy and/or ASCT (57% vs 25%, *P* = 0.049, Fig. 2B).

### Survival analysis of the amyloid and the non-amyloid patients

For the 21 patients with amyloid-related glomerulopathy, presence of anemia and absence of chemotherapy or ASCT were identified as risk factors for death by univariate analysis (Supplementary Table S3, Supplementary Fig. S2A, B). No factor was identified for the risk of ESRD by univariate analysis (Supplementary Table S4).

For the 17 patients with nonamyloid-related glomerulopathy, no factor was identified for the risk of death by univariate analysis (Supplementary Table S5). High eGFR [HR = 0.869; 95% CI 0.869, 0.997, *P* < 0.05] and High hemoglobin [HR = 0.895; 95% CI 0.827, 0.969, *P* < 0.05] were identified as protective factors for ESRD by univariate analysis. Chemotherapy and/or ASCT was not found to be associated with ESRD by univariate survival analysis (Supplementary Table S6).

## Discussion

IgM-MGRS is an uncommon variety of a rare disease with a distinct presentation and natural history and has been increasingly recognized only over the last decade. There is relatively limited clinical literature, especially with respect to the choice of therapies. In this study, the clinicopathologic characteristics of an overall case series of 38 patients treated at our institution were presented. IgM-MGRS is associated a wide spectrum of renal pathology that are quite distinct in their pathogenesis and clinical presentation. The most common renal lesion was amyloidosis, followed by cryoglobulinemic glomerulonephritis. Small proportion presented as proliferative GN with monoclonal IgM deposits, LCDD and C3GN. This was consistent with the results of two previous studies on monoclonal IgM-secreting B-cell lymphoproliferative disorders^5,6^. Amyloidosis was also the most common renal pathological change in patients with IgG-MGRS and IgA-MGRS^4^. A previous study about MGRS with nonamyloid-related glomerulopathy reported that LCDD was the most prevalent histology; however, only 2% of patients were IgM-MGRS^7^.

The clinical features of patients with IgM-MGRS were different from those with IgG-MGRS and IgA-MGRS. In line with the result of the present study, our previous study found that patients with IgM-MGRS (all 11 patients were included in this current study) were more likely to have renal dysfunction, hematuria, anemia and hypocomplementemia compared with those with IgG-MGRS and IgA-MGRS^4^. Comparing IgM-MGRS with amyloidosis and non-amyloidosis, it was revealed that non-amyloid patients were more common to have renal dysfunction, hypertension, hematuria, anemia and hypocomplementemia, which might contribute to the difference between IgM-MGRS and IgG/IgA-MGRS. In the patients with IgM-MGRS, hypocomplementemia was primarily associated with decreases in serum C3 level. However, the decrease in serum C4 level was only found in the non-amyloid patients. Another study found that low serum C4 was more common than low serum C3 in patients with Waldenström macroglobulinemia and other IgM-producing B cell lymphoproliferative disorders^5^. Patients with IgG-MGRS and IgA-MGRS were more likely to have the λ light chain type of M protein. However, in our study κ light chain was predominant due to the contribution of non-amyloid patients, although λ light chain was still more common in the amyloid patients.

In our study, patients with amyloidosis were more often treated with chemotherapy and/or ASCT than those with non-amyloidosis, which was in line with the previous reports^8,9^. We found that patients who did not receive chemotherapy were older, lower eGFR and less organ involvement. The underlying hematologic condition of MGRS (nonmalignant or premalignant stage) and the combination of advanced age and less organ involvement, were probable reasons to not commence chemotherapy in these patients. Treatment with chemotherapy/ASCT is recommended in MGRS patients to prolong survival and preserve renal function^10^. Our study confirmed chemotherapy and/or ASCT benefited survival for amyloid patients, but not the whole cohort. We found chemotherapy and/or ASCT improved renal prognosis for the whole cohort, but not for amyloid or non-amyloid subgroup, which may due to the small number of patients/events and chemotherapy/ASCT was only given in 35% of patients with non-amyloidosis. Bortezomib-based therapy was available in eleven patients. We did not detect the effect of bortezomib-based therapy on overall and renal survival (data not shown), in accordance with the previous study with nonamyloid-related glomerulopathy^7^. However, improved renal survival following bortezomib has previously been observed in other MGRS studies^11–16^. A prospective study comparing bortezomib-based therapy to other chemotherapy would be valuable in clarifying therapeutic options.

There are two findings in this study that deserve attention. First, we found that the amyloid patients seemed have higher mortality and lower incidence of ESRD, compared to the non-amyloid patients. The high mortality for IgM-MGRS with amyloidosis may be due to high frequency of systemic manifestation, including the vital organs of heart and liver. Most of the non-amyloid IgM-MGRS are limited renal injury, thus suggesting that IgM-MGRS patients with non-amyloidosis are surviving on long term renal replacement therapy. Second, we found deep hematologic responses (≥ VGPR) benefited the renal survival of patients with IgM-MGRS, and previous studies have demonstrated that achieving excellent hematologic response in MGRS patients is associated with an improved renal survival^6,12,14,17–19^. As MGRS diagnosis requires renal biopsy, and the renal disease duration before renal biopsy was shorter in the responders in our study, early diagnosis of IgM-MGRS might be essential. Suppression of monoclonal Ig secretion with chemotherapy is required to reach hematologic response and preserve renal outcomes^20^.

This study had some limitations. It is a retrospective study with a small sample size, although this represents the largest series of patients with IgM-MGRS to the best of our knowledge. Those patients in the present study could not represent all IgM-MGRS patients very well, since we excluded patients without kidney biopsy and some amyloidosis can be diagnosed by other means such as by bone marrow biopsy sample or fat aspirate. Limited numbers of patients with IgM-MGRS with nonamyloid-related glomerulopathy received chemotherapy/ASCT. A further study would be needed to explore whether chemotherapy would help prevent ongoing renal damage in this sub-group.

In conclusion, this study included a large group of IgM-MGRS patients, of whom had a diverse variety of clinicopathological features and outcomes. Chemotherapy and/or ASCT and deep hematologic responses might improve renal prognosis.

## Methods

### Patients

We searched all patients at the National Clinical Research Center of Kidney Diseases, Jinling Hospital, Nanjing, China, from 2009 to 2019. The inclusion criteria were as follows: (1) detectable serum monoclonal IgM by immunofixation, (2) performance of a bone marrow biopsy and kidney biopsy, and (3) biopsy-proven MGRS. We excluded patients who were diagnosed with hematological malignancies and required immediate treatment for the clonal disease, including Waldenström macroglobulinemia, multiple myeloma/plasmacytoma, lymphoma or leukemia and patients who had monoclonal gammopathy of undetermined significance and kidney diseases unrelated to M protein.

### Clinical data and pathological findings

Demographic, clinical, laboratory, treatment information and outcomes were obtained retrospectively from the electronic medical records. The kidney biopsy samples were processed as previously reported^4^. Electron microscopy was performed in all biopsy samples, and in 3 cases, glomeruli were not present.

The eGFR was calculated using the Chronic Kidney Disease Epidemiology Collaboration 2009 formula (CKD-EPI). Nephrotic syndrome was defined as 24-h urinary protein excretion over 3.5 g and serum albumin level less than 3 g/dl; anemia was defined as hemoglobin (Hb) less than 11 g/dl; hypertension was defined as systolic blood pressure (BP) ≥ 140 mm Hg, diastolic BP ≥ 90 mm Hg, or treatment with an antihypertensive medication; kidney impairment was defined as serum creatinine over 1.24 mg/dl; hematuria was defined as counts of erythrocytes per microliter over 12; abnormal serum κ/λ FLC ratio was defined as > 1.65 or < 0.26; and hypocomplementemia was defined as serum C3 level < 0.8 g/l or C4 level < 0.1 g/l. The definition of cardiac and liver involvement in amyloid patients are based on National Comprehensive Cancer Network guideline. Decreased blood pressure was defined by systolic blood pressure decreased more than 20 mmHg than patients’ baseline blood pressure or anti-hypertensive agents were no longer used to control blood pressure in previous hypertensive patients.

### Diagnostic, response criteria and follow‑up

Two kidney pathologists and one hematopathologist reviewed the kidney and bone marrow biopsies. Consensus international criteria were used for the diagnoses of MGRS^1^. Computed tomography, with or without positron-emission tomography, was performed in all patients. We collected clinical and laboratory data at the time of kidney biopsy, after upfront treatment, and at last follow-up. Hematologic and kidney response was defined according to the 2012 International Society of Amyloidosis Criteria for amyloidosis and the 2013 Consensus criteria from the Sixth International Workshop on Waldenström Macroglobulinemia^21,22^. Overall and kidney survival rates were calculated from the time of kidney biopsy to the last follow-up (by June 30, 2020), end stage renal disease (ESRD), or death. ESRD was defined as eGFR of less than 15 ml/(min·1.73 m^2^) for at least 3 months or required maintenance dialysis or kidney transplant.

### Statistical analysis

Analysis was performed with the SPSS version 19.0 statistical software package (SPSS, Chicago, IL). Continuous data were expressed as means with standard deviations (SDs) or as medians with interquartile ranges (IQRs). Categorical data were presented as proportions. Patients were divided into two groups on the basis of their kidney pathology: amyloid and non-amyloid. Differences between groups were compared using t-test for normally distributed and homogeneous quantitative data, Mann–Whitney *U* test for non-normally distributed, heterogeneous quantitative and semiquantitative data, and Fisher’s exact test for polychotomous data. Patient survival and renal survival were estimated by the Kaplan–Meier method. Kaplan–Meier analysis was used to compare survival between amyloid and non-amyloid patients. Possible indicators for death and renal endpoint were examined by univariate analysis (log-rank test and Cox post hoc for calculation of hazard ratio, HR). The small number of patients/events did not permit multivariate analysis of overall survival or renal survival. All tests were two-sided, and *P* < 0.05 indicated statistical significance.

### Ethical approval and informed consent

The research was in compliance with the Declaration of Helsinki and approved by the Ethics Committee of Jinling Hospital (2020NZKYKS-002-01). As this was a retrospective observational study with full patient anonymity, the requirement for written informed consent was waived.

## Supplementary Information

Supplementary Information.

## Data Availability

The datasets are available from the corresponding author on reasonable request.

## References

[1] Leung N (2012). Monoclonal gammopathy of renal significance: When MGUS is no longer undetermined or insignificant. Blood.

[2] Castillo JJ, Callander NS, Baljevic M, Sborov DW, Kumar S (2021). The evaluation and management of monoclonal gammopathy of renal significance and monoclonal gammopathy of neurological significance. Am. J. Hematol..

[3] Leung N, Bridoux F, Nasr SH (2021). Monoclonal gammopathy of renal significance. N. Engl. J. Med..

[4] Liang D (2020). Types of M protein and clinicopathological profiles in patients with monoclonal gammopathy of renal significance. J. Nephrol..

[5] Higgins L (2018). Kidney involvement of patients with waldenstrom macroglobulinemia and other IgM-producing B cell lymphoproliferative disorders. Clin. J. Am. Soc. Nephrol..

[6] Chauvet S (2015). Kidney diseases associated with monoclonal immunoglobulin M-secreting B-cell lymphoproliferative disorders: A case series of 35 patients. Am. J. Kidney Dis..

[7] Khera A (2019). Long term outcomes in monoclonal gammopathy of renal significance. Br. J. Haematol..

[8] Yu XJ (2020). Renal pathologic spectrum and clinical outcome of monoclonal gammopathy of renal significance: A large retrospective case series study from a single institute in China. Nephrology.

[9] Pozzi C (2003). Light chain deposition disease with renal involvement: Clinical characteristics and prognostic factors. Am. J. Kidney Dis..

[10] Fermand JP (2013). How I treat monoclonal gammopathy of renal significance (MGRS). Blood.

[11] Cohen C (2015). Bortezomib produces high hematological response rates with prolonged renal survival in monoclonal immunoglobulin deposition disease. Kidney Int..

[12] Sayed RH (2015). Natural history and outcome of light chain deposition disease. Blood.

[13] Li XM (2016). Clinicopathological characteristics and outcomes of light chain deposition disease: An analysis of 48 patients in a single Chinese center. Ann. Hematol..

[14] Chauvet S (2017). Treatment of B-cell disorder improves renal outcome of patients with monoclonal gammopathy-associated C3 glomerulopathy. Blood.

[15] Ziogas DC (2017). Hematologic and renal improvement of monoclonal immunoglobulin deposition disease after treatment with bortezomib-based regimens. Leuk. Lymphoma.

[16] Huang X (2014). Induction therapy with bortezomib and dexamethasone followed by autologous stem cell transplantation versus autologous stem cell transplantation alone in the treatment of renal AL amyloidosis: A randomized controlled trial. BMC Med..

[17] Kourelis TV (2016). Outcomes of patients with renal monoclonal immunoglobulin deposition disease. Am. J. Hematol..

[18] Mohan M (2017). Clinical characteristics and prognostic factors in multiple myeloma patients with light chain deposition disease. Am. J. Hematol..

[19] Vignon M (2017). Current anti-myeloma therapies in renal manifestations of monoclonal light chain-associated Fanconi syndrome: A retrospective series of 49 patients. Leukemia.

[20] Bridoux F (2015). Diagnosis of monoclonal gammopathy of renal significance. Kidney Int..

[21] Palladini G (2012). New criteria for response to treatment in immunoglobulin light chain amyloidosis based on free light chain measurement and cardiac biomarkers: Impact on survival outcomes. J. Clin. Oncol..

[22] Owen RG (2013). Response assessment in Waldenstrom macroglobulinaemia: Update from the VIth international workshop. Br. J. Haematol..

