# Supplementary material for: Clinicopathologic characteristics and prognostic analysis of monoclonal gammopathy of renal significance (MGRS) in patients with IgM monoclonal gammopathy: a case series
Source: Sci Rep. 2022 Oct 10;12:16994. doi: 10.1038/s41598-022-21152-0 (PMC9551084; doi:10.1038/s41598-022-21152-0)
Supplement: Supplementary file 1 — Supplementary Information. [file 41598_2022_21152_MOESM1_ESM.docx]

**Clinicopathologic Characteristics and Prognostic Analysis of Monoclonal Gammopathy of Renal Significance (MGRS) in Patients with IgM Monoclonal Gammopathy: A Case Series**

Jing Liu^#^, Dandan Liang^#^, Shaoshan Liang, Feng Xu, Xianghua Huang, Song Jiang*, Jinhua Hou*

**Supplementary Information**

**Supplementary Table S1: Renal Histopathological characteristics in patients with MGRS with IgM Monoclonal Gammopathy**

| **Characteristics** | **Total** | **Amyloid** | **Non-amyloid**^*^ | ***P*-value** |
| --- | --- | --- | --- | --- |
| N | 38 | 21 | 17 |  |
| **Light microscopy** |  |  |  |  |
| Glomerular sclerosis% (IQR) | 8.1(0-15.2) | 6.2(0.9-13.9) | 8.1(0-29.6) | 0.636 |
| Acute tubular injury, n (%) | 24(63.2) | 11(52.4) | 13(76.5) | 0.181 |
| Tubulointerstitial fibrosis |  |  |  | 0.496 |
| 0~25%, n (%) | 24(63.2) | 13(61.9) | 11(64.7) |  |
| 25%~50%, n (%) | 13(34.2) | 8(38.1) | 5(38.5) |  |
| >50%, n (%) | 1(2.6) | 0 | 1(5.9) |  |
| Interstitial inflammation |  |  |  | 0.190 |
| 0~25%, n (%) | 7(18.4) | 6(28.6) | 1(5.9) |  |
| 25%~50%, n (%) | 20(52.6) | 9(42.9) | 11(64.7) |  |
| >50%, n (%) | 11(28.9) | 6(28.6) | 5(29.4) |  |
| Arteriosclerosis, n (%) | 27(71.1) | 15(71.4) | 12(70.6) | 1 |
| **Immunofluorescence** |  |  |  |  |
| κ light chain, n (%) |  | 5(23.8) | 11(64.7) ^#^ | 0.020 |
| λ light chain, n (%) |  | 16(76.2) | 6(35.3) ^#^ |  |

^*^ 12 patients with cryoglobulinemic GN, 3 with proliferative GN with monoclonal IgM deposits, 1 with light-chain deposition disease, and 1 with C3 GN.

^#^ The staining of κ and λ light chain were both negative in 1 patient with C3 GN, and both positive in 1 patient with cryoglobulinemic GN.

**Supplementary Table S2: Compared baseline characteristics of patients treated with no chemotherapy and** **chemotherapy and/or ASCT**

| **Characteristics** | **No** **chemotherapy** | **Chemotherapy and/or ASCT** | ***P*-value** |
| --- | --- | --- | --- |
| N | 14 | 24 |  |
| Renal pathological features |  |  |  |
| Amyloid/Non-amyloid | 3/11 | 18/6 | 0.002 |
| Age (IQR), years | 64 (51-68) | 61 (53-68) | 0.800 |
| Sex, men/women | 10/4 | 16/8 | 1 |
| Hypertension, n (%) | 10 (71) | 8 (33) | 0.042 |
| Cardiac involvement, n (%) | 2 (14) | 12 (50) | 0.039 |
| Liver involvement, n (%) | 0 | 3 (13) | 0.283 |
| Hematuria, n (%) | 9 (64) | 10 (42) | 0.313 |
| Serum creatinine (IQR), mg/dl | 1.67 (1.09-2.77) | 0.94 (0.78-1.86) | 0.037 |
| eGFR (IQR), ml/min per 1.73 m^2^ | 39.9 (19.9-68.8) | 77.3 (36.6-87.1) | 0.058 |
| Kidney impairment, n (%) | 10 (71) | 9 (38) | 0.091 |
| Edema, n (%) | 13 (93) | 19 (79) | 0.383 |
| Proteinuria (IQR), g/d | 4.80 (1.21-9.64) | 3.53 (2.00-6.14) | 0.676 |
| Serum albumin (IQR), g/l | 29.2 (23.3-35.7) | 29.8 (25.7-36.1) | 0.643 |
| Nephrotic syndrome, n (%) | 7 (50) | 9 (38) | 0.510 |
| Hemoglobin (IQR), g/l | 98 (78-111) | 118 (105-133) | 0.002 |
| Anemia, n (%) | 11 (79) | 7 (29) | 0.006 |
| Light-chain type, n (%) |  |  | 0.181 |
| κ | 10 | 11 |  |
| λ | 4 | 13 |  |
| Abnormal serum κ/λ FLC ratio, n (%) | 8 (57) | 10 (42) | 0.503 |
| Low serum complement, n (%) | 8 (57) | 7 (29) | 0.168 |
| Serum IgM (IQR), g/l | 4.91 (2.78-14.28) | 6.44 (2.98-10.53) | 1 |
| Renal disease duration before renal biopsy (IQR), months | 8.9 (4.3-25.0) | 4.2 (2.4-12.5) | 0.152 |

**Abbreviations:** IQR, interquartile range; FLC, free light chain, ASCT, autologous peripheral blood stem cell transplantation.

**Supplementary Table S3: Univariate analysis of amyloid patients for death**

|  | **HR** | **95% CI** | | ***P*-value** |
| --- | --- | --- | --- | --- |
|  |  | **Lower** | **Upper** |  |
| **Univariate analysis** |  |  |  |  |
| Age (years) | 1.036 | 0.955 | 1.125 | 0.394 |
| Gender (male vs female) | 3.785 | 0.465 | 30.805 | 0.213 |
| Serum creatinine (mg/dl) | 1.263 | 0.561 | 2.846 | 0.573 |
| eGFR (ml/min/1.73m^2^) | 0.985 | 0.963 | 1.007 | 0.170 |
| KI (yes vs no) | 2.786 | 0.656 | 11.822 | 0.165 |
| Proteinuria (g/d) | 0.976 | 0.763 | 1.248 | 0.848 |
| Serum albumin (g/l) | 0.919 | 0.823 | 1.027 | 0.138 |
| IgM, g/l | 0.985 | 0.905 | 1.072 | 0.728 |
| Light-chain type (κ vs λ) | 0.689 | 0.167 | 2.854 | 0.608 |
| Cardiac involvement (yes vs no) | 0.749 | 0.179 | 3.141 | 0.693 |
| Liver involvement (yes vs no) | 1.410 | 0.279 | 7.119 | 0.677 |
| Low serum complement (yes vs no) | 5.551 | 0.577 | 53.454 | 0.138 |
| Abnormal serum κ/λ FLC ratio (yes vs no) | 2.233 | 0.553 | 9.014 | 0.259 |
| Hb (g/l) | 0.965 | 0.928 | 1.004 | 0.080 |
| Anemia (yes vs no) | 4.949 | 1.090 | 22.477 | 0.038 |
| Chemotherapy and/or ASCT (yes vs no) | 0.110 | 0.018 | 0.667 | 0.016 |

**Abbreviations:** HR, hazard ratio; CI, confidence interval; eGFR, estimated glomerular filtration rate; KI, kidney impairment; FLC, free light chain; Hb, hemoglobin; ASCT, autologous peripheral blood stem cell transplantation.

**Supplementary Table S4: Univariate analysis of amyloid patients for ESRD**

|  | **HR** | **95% CI** | | ***P*-value** |
| --- | --- | --- | --- | --- |
|  |  | **Lower** | **Upper** |  |
| **Univariate analysis** |  |  |  |  |
| Age (years) | 0.957 | 0.859 | 1.066 | 0.421 |
| Gender (male vs female) | 1.921 | 0.159 | 23.224 | 0.608 |
| Serum creatinine (mg/dl) | 1.732 | 0.584 | 5.137 | 0.322 |
| eGFR (ml/min/1.73m^2^) | 0.987 | 0.952 | 1.023 | 0.467 |
| KI (yes vs no) | 6.359 | 0.555 | 72.818 | 0.137 |
| Proteinuria (g/d) | 0.886 | 0.561 | 1.398 | 0.602 |
| Serum albumin (g/l) | 1.005 | 0.837 | 1.206 | 0.959 |
| IgM, g/l | 1.140 | 0.986 | 1.317 | 0.076 |
| Light-chain type (κ vs λ) | 2.048 | 0.173 | 24.305 | 0.570 |
| Cardiac involvement (yes vs no) | 0.409 | 0.034 | 4.889 | 0.480 |
| Liver involvement (yes vs no) | 2.313 | 0.183 | 29.167 | 0.517 |
| Low serum complement (yes vs no) | 0.046 | 0.000 | 9.815E+15 | 0.880 |
| Abnormal serum κ/λ FLC ratio (yes vs no) | 1.383 | 0.119 | 16.061 | 0.795 |
| Hb (g/l) | 0.975 | 0.912 | 1.042 | 0.455 |
| Anemia (yes vs no) | 4.472 | 0.279 | 71.807 | 0.290 |
| Chemotherapy and/or ASCT (yes vs no) | 0.101 | 0.006 | 1.612 | 0.105 |

**Abbreviations:** ESRD, end stage renal disease; HR, hazard ratio; CI, confidence interval; eGFR, estimated glomerular filtration rate; KI, kidney impairment; FLC, free light chain; Hb, hemoglobin; ASCT, autologous peripheral blood stem cell transplantation.

**Supplementary Table S5: Univariate analysis of non-amyloid patients for death**

|  | **HR** | **95% CI** | | ***P*-value** |
| --- | --- | --- | --- | --- |
|  |  | **Lower** | **Upper** |  |
| **Univariate analysis** |  |  |  |  |
| Age (years) | 1.098 | 0.963 | 1.251 | 0.163 |
| Gender (male vs female) | 40.544 | 0.013 | 131423.627 | 0.369 |
| Serum creatinine (mg/dl) | 120.459 | 0.093 | 156659.085 | 0.190 |
| eGFR (ml/min/1.73m^2^) | 0.854 | 0.703 | 1.037 | 0.111 |
| KI (yes vs no) | 63.570 | 0.007 | 566427.712 | 0.371 |
| Proteinuria (g/d) | 1.858 | 0.759 | 4.548 | 0.175 |
| Serum albumin (g/l) | 0.921 | 0.814 | 1.043 | 0.196 |
| IgM, g/l | 0.797 | 0.552 | 1.151 | 0.226 |
| Light-chain type (κ vs λ) | 2.550 | 0.281 | 23.116 | 0.405 |
| Low serum complement (yes vs no) | 40.544 | 0.013 | 131423.627 | 0.369 |
| Abnormal serum κ/λ FLC ratio (yes vs no) | 1.871 | 0.117 | 29.956 | 0.658 |
| Hb (g/l) | 0.961 | 0.906 | 1.020 | 0.192 |
| Anemia (yes vs no) | 62.044 | 0.012 | 333707.994 | 0.346 |
| Chemotherapy and/or ASCT (yes vs no) | 1.646 | 0.231 | 11.743 | 0.619 |

**Abbreviations:** HR, hazard ratio; CI, confidence interval; eGFR, estimated glomerular filtration rate; KI, kidney impairment; FLC, free light chain; Hb, hemoglobin; ASCT, autologous peripheral blood stem cell transplantation.

**Supplementary Table S6: Univariate analysis of non-amyloid patients for renal endpoint**

|  | **HR** | **95% CI** | | ***P*-value** |
| --- | --- | --- | --- | --- |
|  |  | **Lower** | **Upper** |  |
| **Univariate analysis** |  |  |  |  |
| Age (years) | 1.085 | 0.975 | 1.208 | 0.135 |
| Gender (male vs female) | 1.782 | 0.360 | 8.826 | 0.479 |
| Serum creatinine (mg/dl) | 1.701 | 1.133 | 2.555 | 0.010 |
| eGFR (ml/min/1.73m^2^) | 0.931 | 0.869 | 0.997 | 0.041 |
| KI (yes vs no) | 35.348 | 0.026 | 47738.699 | 0.332 |
| Proteinuria (g/d) | 1.088 | 0.940 | 1.259 | 0.258 |
| Serum albumin (g/l) | 0.948 | 0.859 | 1.046 | 0.284 |
| IgM, g/l | 1.026 | 0.824 | 1.278 | 0.817 |
| Light-chain type (κ vs λ) | 1.379 | 0.272 | 6.983 | 0.698 |
| Low serum complement (yes vs no) | 3.859 | 0.467 | 31.868 | 0.210 |
| Abnormal serum κ/λ FLC ratio (yes vs no) | 1.413 | 0.284 | 7.034 | 0.673 |
| Hb (g/l) | 0.895 | 0.827 | 0.969 | 0.006 |
| Anemia (yes vs no) | 35.632 | 0.057 | 22271.354 | 0.277 |
| Chemotherapy and/or ASCT (yes vs no) | 0.645 | 0.156 | 2.660 | 0.544 |

**Abbreviations:** ESRD, end stage renal disease; HR, hazard ratio; CI, confidence interval; eGFR, estimated glomerular filtration rate; KI, kidney impairment; FLC, free light chain; Hb, hemoglobin; ASCT, autologous peripheral blood stem cell transplantation.

**Supplementary Figure S1: A**: Comparing survival of IgM-MGRS patients with responders (≥VGPR) and non-responders (<VGPR).

**B:** Comparing renal survival of IgM-MGRS patients with responders (≥VGPR) and non-responders (<VGPR).

**Abbreviations:** MGRS, monoclonal gammopathy of renal significance; VGPR, a very good partial response.

**Supplementary Figure S2.** **A:** Comparing survival of IgM-MGRS patients with amyloid-related glomerulopathy with and without anemia.

**B:** Comparing survival of IgM-MGRS patients with amyloid-related glomerulopathy with chemotherapy and/or ASCT and without chemotherapy and/or ASCT.

**Abbreviations:** MGRS, monoclonal gammopathy of renal significance; ASCT, autologous peripheral blood stem cell transplantation.
